# Wider determinants of adverse birth outcomes in Birmingham and Solihull

**DOI:** 10.3389/fpubh.2025.1544903

**Published:** 2025-04-15

**Authors:** David Ellis, Chung Him Au-Yeung, Alexander Dallaway, Ranjana Basra, Sylvia Owusu-Nepaul, Jenny Riley, Rebecca Howell-Jones, Justin Varney, Marion Gibbon

**Affiliations:** ^1^Public Health, Birmingham City Council, Birmingham, United Kingdom; ^2^Faculty of Education, Health and Wellbeing, School of Health and Society, University of Wolverhampton, Wolverhampton, United Kingdom; ^3^Warwickshire Institute for the Study of Diabetes, Endocrinology and Metabolism (WISDEM), University Hospitals Coventry and Warwickshire NHS Trust, Coventry, United Kingdom; ^4^Birmingham Women's and Children's Hospital Foundation Trust, Birmingham, United Kingdom

**Keywords:** pregnancy outcome, pregnancy complications, health inequities, social determinants of health, socioeconomic factors, ethnicity, logistic models

## Abstract

**Introduction:**

Birmingham and Solihull face significant challenges related to adverse birth outcomes. This study aimed to identify demographic, socioeconomic, and lifestyle factors associated with an increased risk of low birth weight, premature birth, stillbirth, and neonatal death in Birmingham and Solihull.

**Methods:**

Births (*n* = 41, 231) between October 2020 and April 2023 were analysed. The attributable fraction of premature births and low birth weight (LBW) attributable to socioeconomic and ethnic inequality was calculated. Multiple logistic regression analyses identified groups that had increased odds of premature birth (*n* = 3, 312), LBW (*n* = 1, 197), stillbirth (*n* = 173), and neonatal death (*n* = 208).

**Results:**

Attributable fraction analysis estimated that 191 premature births and 211 LBWs each year would not have occurred if all women had the same rates as White women living in the least deprived areas. Ethnicity, socioeconomic deprivation, medical care, lifestyle, and vulnerability status were found to be significant risk factors for adverse birth outcomes. Asian and Black women had 1.4–2.7 times the odds of LBW compared to White women (*p* < 0.01). Black women had increased odds of stillbirth (OR : 1.75, *p* = 0.017) and Asian women had increased odds of neonatal death (OR : 1.90, *p* < 0.001). The odds of LBW (OR : 3.3), premature birth (OR : 27.2), and neonatal death (OR : 5.6) were significantly increased for twins (*p* < 0.001). For women smoking at delivery, the odds of LBW (OR : 2.3), premature birth (OR : 1.5), and stillbirth (OR : 1.6) were significantly increased (*p* < 0.05). Deprivation, and/or financial and housing issues also increased the odds of adverse birth outcomes (*p* < 0.05).

**Discussion:**

These findings underscore the importance of targeted interventions and support for at-risk populations to reduce adverse birth outcomes in vulnerable communities.

## 1 Introduction

Birmingham and Solihull (BSol) Integrated Care system covers two different local authority areas. Overall, BSol faces substantial challenges related to adverse pregnancy outcomes. The rates of all key pregnancy outcomes are significantly higher in BSol than the England average including for low birth weight (LBW) (35.8% higher), premature birth (11.3% higher), stillbirths (27.7% higher), and neonatal mortality (97.9% higher) (1). The rates of these adverse pregnancy outcomes have been significantly higher in BSol for over a decade (see Supplementary Figure S1). These increased rates are largely dominated by Birmingham's contribution. The rates of each of these outcomes are higher in Birmingham than in Solihull. In addition to the greater population size of Birmingham, the birth rate in Birmingham is much higher than in Solihull. In 2021, Birmingham City made up 87% of the births in BSol (1).

Birmingham suffers from high levels of deprivation and has been ranked as the 7th most deprived local authority in England with 43% of the city's population living in the most deprived 10% of neighbourhoods in England. This increases to 51% for those aged 0–15 years indicating that children are disproportionately affected by deprivation (2). In contrast, Solihull is the 32nd least deprived upper-tier local authority with 12% of the population living in the most deprived 10% of neighbourhoods in England. However, there is significant polarisation in Solihull with over half of North Solihull residents living in the most deprived 10% of neighbourhoods in England (3). Socioeconomic deprivation is known to be a significant risk factor for negative birth outcomes. Meta-analyses of maternal outcomes in the UK have found that women living in the most deprived quintiles have significantly higher rates of LBW, premature birth, stillbirth, and neonatal death than women living in the least deprived areas (4–6). Additionally, between 2014 and 2017 in England, there was an unexpected rise in infant mortality rate. One study estimated that around a third of the increase could be attributed to the simultaneous rise in child poverty (7).

In 2020-22, the most deprived 10% of counties and unitary authorities in England had an average infant mortality rate of 6.0 per 1,000 live births compared to 2.8 for those living in the 10% least deprived (1). BSol's infant mortality rate was higher than average for the most deprived areas at 6.9 per 1,000 live births. This suggests that there may be additional inequalities contributing to the increased infant mortality rate.

Birmingham is the most ethnically diverse core city in England and Wales.^1^ In the 2021 census, 51.5% of BSol residents described themselves as something other than “White British”. After the White British group (48.5%), the largest groups were Pakistani (14.9%), Indian (5.7%), Black African (5.0%), and Other White (3.8%) (8). Studies in the UK have found significant variations in infant mortality rates between ethnic groups, with Pakistani and Caribbean infants typically suffering the highest rates (9, 10). Asian, Black, and Mixed ethnicity residents of Birmingham also experience other health inequalities such as increased risk of type 2 diabetes (11).

The impact of socioeconomic and ethnic inequalities on the risk of adverse pregnancy outcomes may be cumulative. The interaction of belonging to a minoritised ethnic group and living in a deprived area may increase the risk of adverse birth outcomes. This would mean those from a higher-risk ethnic group who live in a deprived area are at greater risk than those of the same ethnic group in a less deprived area. Similarly, those in a high-risk ethnic group are at greater risk than other groups living in areas of the same deprivation level. South Asian women and Black women living in the most socioeconomically deprived areas were found to be at the highest risk of stillbirth, premature birth and fetal growth restriction (12). This is particularly important for Birmingham due to its high levels of deprivation and ethnic diversity.

Studies on the wider determinants of birth outcomes tend to be conducted at a national level (4, 5, 9, 10, 12). Despite having a larger sample size, these studies may generalise issues and mask inequalities that have substantial impacts in some regions and not others. Where studies on maternal health have been conducted in more focused geographical areas, these tend to be qualitative (13, 14). To date, no large-scale quantitative study of the wider determinants of birth outcomes has been performed in BSol. This, however, is key to understanding and developing the local response. The size of the Birmingham and Solihull population (1.36 million) provides a unique opportunity for a place-based analysis of the wider determinants of health (8).

This study aimed to determine (i) the inequalities in adverse birth outcomes associated with ethnicity and social deprivation, and their cumulative impact, and (ii) the key factors associated with an increased odds of adverse birth outcomes for women living in Birmingham and Solihull.

## 2 Methods

Anonymised routine maternity data were obtained for all births (*n* = 41, 231) between October 2020 and April 2023 occurring at four maternity units across Birmingham provided by Birmingham Women's and Children's NHS Foundation Trust (*n* = 19, 152) and University Hospital Birmingham NHS Foundation Trust (*n* = 22, 079).

Routine data included demographic information, health and risk factors, antenatal care and pregnancy outcomes. Demographic data about the women giving birth included age, whether they had financial or housing issues, the Index of Multiple Deprivation (IMD) decile of the Lower Super Output Area (LSOA) that they lived in, and ethnicity. IMD is a national metric in which areas are ranked from most deprived (rank 1) to least deprived. The metric combines data on employment, education, health and disability, crime, barriers to housing, and lived environment (15). Due to the small number of neighbourhoods in IMD quintiles 3, 4, and 5, it was necessary to combine these quintiles into one group (3+). Maternal ethnicity was coded using the Office of National Statistics categorisation system from the UK census. The dataset also included the person's citizenship status, whether they have difficulty understanding English and whether an interpreter was required at antenatal appointments. Information on the person's health and risk factors included sensory and physical disabilities, obesity status, smoking status at delivery, gestational diabetes, consanguineous union, whether the woman was a victim of female genital mutilation or domestic abuse and whether they have learning disabilities. Antenatal care data included how many, if any, antenatal appointments were missed, the gestation of the first booking, and whether folic acid was taken during pregnancy. Pregnancy outcomes included gestation at birth, whether the baby was less than 2500 grams at birth, whether the baby was stillborn, or died within 28 days of birth (neonatal death).

All maternity units used the BadgerNet administrative system (16). Cases were removed from the dataset if they related to a pregnancy involving three or more babies, had an unknown Index of Multiple Deprivation (IMD), or had an unknown number of missed antenatal appointments. For pregnancies involving twins, only the first birth was included to limit women appearing in the data twice. However, since the data were anonymised and spanned over 31 months, it is likely that a small number of women still appear twice by having multiple pregnancies in this period. After these exclusions, there were 39,972 (96.9%) unique births remaining in the data.

Four adverse pregnancy outcomes were investigated. Two intermediate outcomes: i. premature birth, ii. LBW, and two final outcomes: iii. stillbirth, and iv. neonatal death. Premature birth and LBW were defined such that they are mutually exclusive: Premature birth was defined as a live baby born before 37 weeks of gestation, and LBW was defined as a live full-term baby (gestation of 37 weeks or more) weighing less than 2500 grams. Stillbirth is defined by the Births and Deaths Registration Act 1953 as “a child which has issued forth from its mother after the 28th week of pregnancy and which did not at any time after being completely expelled from its mother breathe or show other signs of life” (17). Neonatal death is defined as the death of an infant before the 28th day of life (18).

### 2.1 Statistical analysis

The unadjusted attributable fraction (AF) of LBW and premature births attributed to ethnicity and IMD were estimated using


(1) $$\begin{array}{l} AF=\frac{PR-1}{PR}, \end{array}$$

where *PR* is the outcome prevalence of the exposed group compared to the unexposed group given by


$$\begin{array}{l} PR=\frac{\text{Prevalence of outcome in the exposed}}{\text{Prevalence of outcome in the unexposed}}. \end{array}$$

The attributable fractions for stillbirth and neonatal death were not calculated due to the small number of outcomes. For the AF calculation, an additional 173 cases (0.5%) were removed since the person's ethnicity was unknown. The remaining births were then aggregated into broader ethnic groups: Asian, Black, Middle Eastern, Mixed, Other and White. The “unexposed” reference population is taken to be White women living in areas with an IMD quintile of 3 or higher. The 95% confidence interval is calculated using bootstrapping with 1,000 bootstrap distributions for each outcome variable. The AF was calculated using a locally written Python library called EquiPy.

A multiple logistic regression analysis was performed for each of the four outcomes to examine the odds associated with a set of determinants of health, considering variables beyond ethnicity and IMD. This makes it possible to explore multifaceted relationships between several explanatory factors and birth outcomes. For premature birth and LBW, the following variables were included in the regression: ethnicity, IMD quintile, financial/housing issues, substance abuse status, social services involvement, the mother's age group, whether the baby was a twin, mental health issues, sensory/physical disability, body mass index (BMI)>35, gestational diabetes, smoking at delivery, folic acid taken during pregnancy, late antenatal booking (more than 20 weeks after conception), 4 or more missed appointments [half of the number recommended the WHO (19)], and consanguineous union. The reference group was taken to be White British women, aged 20-29, living in an area with an IMD of 3 or more without known risk factors. For the logistic regressions of final outcomes, fewer explanatory variables were considered due to the smaller number of final outcome occurrences. These were: broad ethnicity (Asian, Black, Middle Eastern, Mixed, Other, White, and unknown), IMD, the mother's age group, folic acid taken during pregnancy, financial/housing issues, whether the baby is a twin, mental health issues and smoking at delivery. Similarly to the regressions for the intermediate outcomes, the reference group is chosen to be White women, aged 20–29, living in an area with an IMD of 3 or more without known risk factors. Regression results are plotted as a forest plot produced using the R package Forestplot (20).

An alpha level of 0.05 was required for statistical significance in all tests. All data processing was performed in R Studio (version 2023.12.1, R version 4.3.3). AF was performed in Python 3.13 while the logistic regression was performed in R Studio (version 2023.12.1, R version 4.3.3). Data visualisations were performed in both R Studio (version 2023.12.1, R version 4.3.3) and Python 3.13. All code used in this study is available on GitHub.

## 3 Results

The number of intermediate and final outcomes were: i. premature birth (*n* = 3, 312), ii. LBW (*n* = 1, 197), iii. stillbirth (*n* = 173), and iv. neonatal death (*n* = 208). The number of stillbirths and neonatal deaths that occurred following a full-term normal birth weight, LBW or premature birth are shown in Supplementary Table S1. A full breakdown of intermediate and final outcomes across each of the considered variables is given in Supplementary Tables S2, S3, respectively. The distribution of births across the five BSol localities^2^ is shown in Supplementary Figure S3. Additionally, a descriptive analysis of the population included in the analysis, including trends in risk factors across ethnicity and IMD is given in Supplementary Section 3.

**Figure 1:**
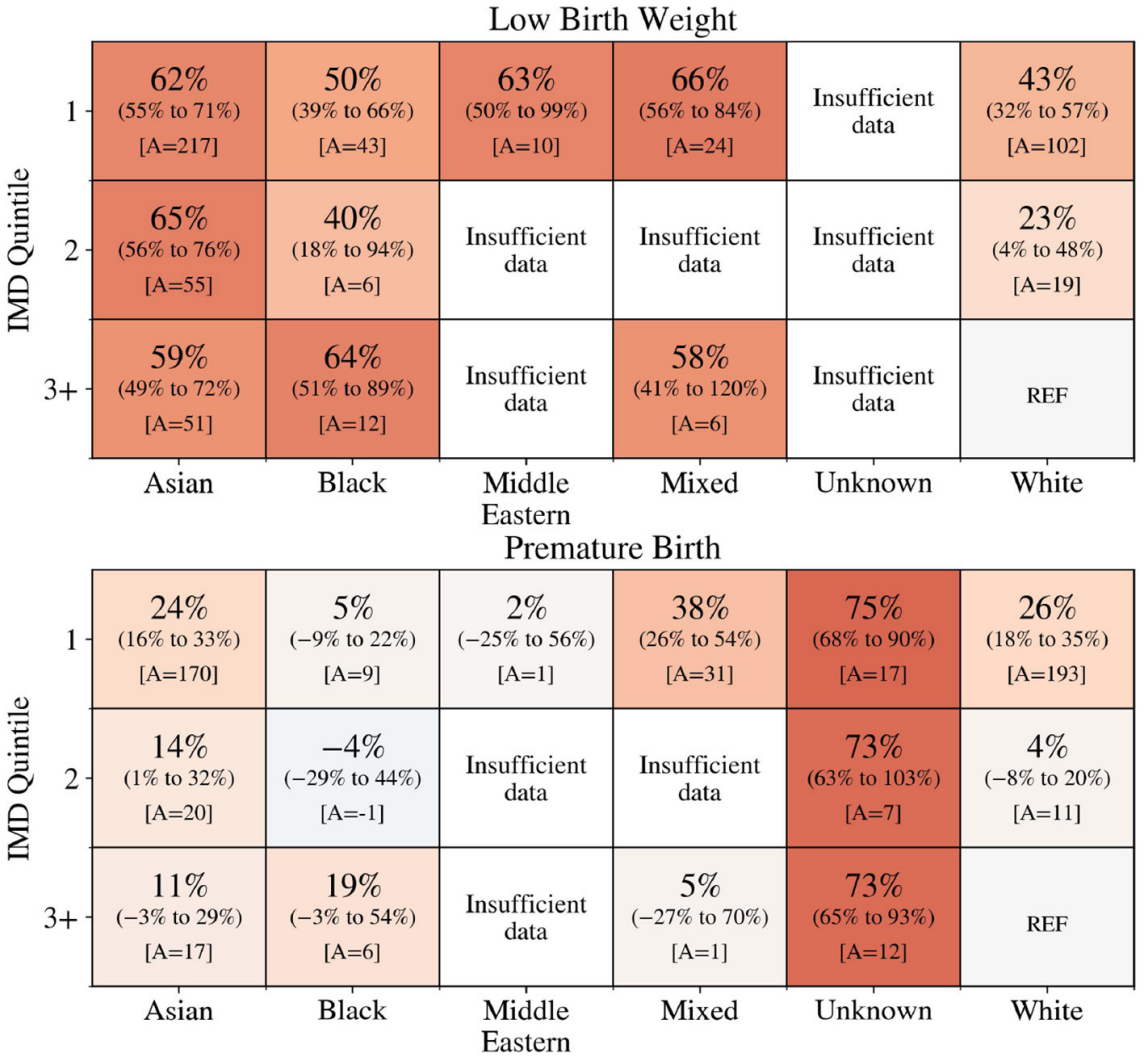
Percentage of low birth weight **(top)** and premature births **(bottom)** across ethnicity and IMD that would not have occurred if the woman had the same level of risk as White ethnicity women in the least deprived area (IMD quintile 3+). A is the number of low birth weight **(top)** and premature births **(bottom)** that would have been avoided. The colour reflects the attributable fraction from negative (blue) to positive (orange).

The unadjusted AF of LBW and premature births attributable to the woman's ethnicity and IMD was calculated. Figure 1 shows that almost all groups, for which there were sufficient data, experienced a higher rate of premature and LBW than the reference group. Results for Other ethnicities are not shown due to limited records. Furthermore, the AF was higher for women living in the most deprived areas who were Asian (LBW: 62%, 95% CI = [55%; 71%]; premature birth: 24% [16%, 33%]), Mixed ethnicity (premature birth: 38% [26%, 54%]) and White (LBW: 43% [32%, 57%]; premature birth: 26% [18%, 35%]). For premature birth, those with an unknown ethnicity had the highest attributable fraction. Overall, it is estimated that if all women had the same prevalence as the reference group there would have been 211 fewer low-weight births each year from an average total of 463, and 191 fewer premature births each year from an average total of 1,282.

**Figure 2:**
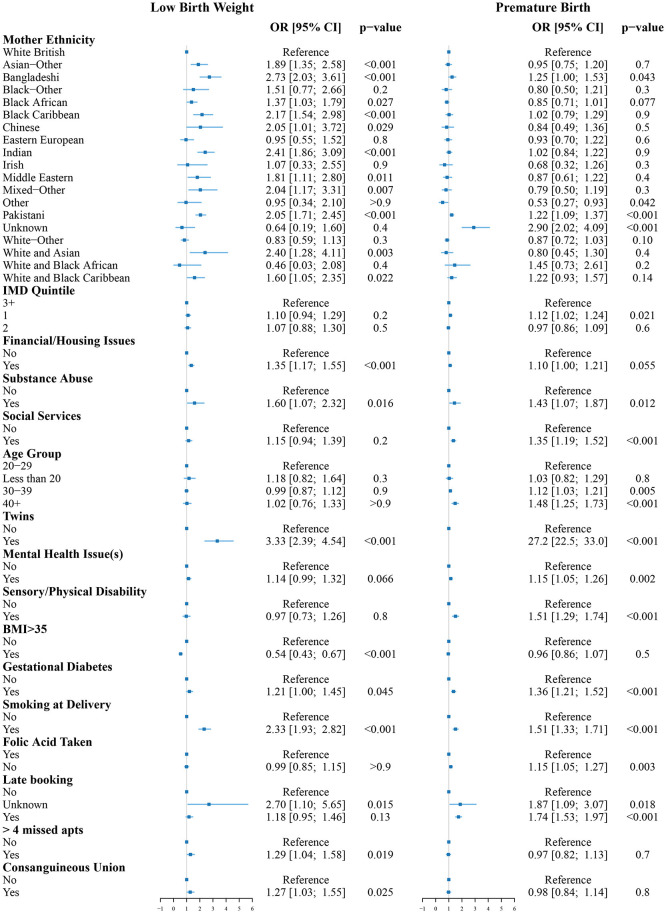
Odds ratios for low birth weight **(left)** and premature birth **(right)** compared to the reference group (White women, aged 20–29, living in an area with IMD of 3 or more without known risk factors). The reference group is indicated by the vertical line at OR = 1.

**Figure 3:**
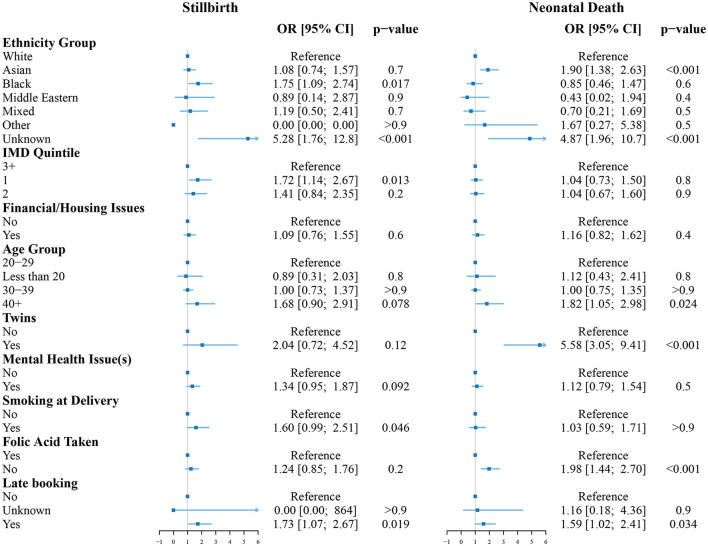
Odds ratios for stillbirth **(left)** and neonatal death **(right)** compared to the reference group (White women, aged 20–29, living in an area with IMD of 3 or more without known risk factors). The reference group is indicated by the vertical line at OR = 1.

The logistic regression results for the two intermediate outcomes, LBW and premature birth, are shown in Figure 2 and the logistic regression results for the two final outcomes, stillbirth and neonatal death are shown in Figure 3. The variance inflation factor for these variables is less than or approximately equal to two, indicating that any correlations between the variables are not large enough to indicate the presence of multicollinearity (Supplementary Figure S2).

The odds of LBW were increased for Asian-Other, Bangladeshi, Black African, Black Caribbean, Chinese, Indian, Middle Eastern, Mixed-Other, Pakistani, or unknown ethnicity women. All had between 1.37 and 2.73 times increased odds of LBW (*p* < 0.05) compared to White British women. Living with financial and/or housing issues significantly increased the odds of LBW (OR = 1.35 [1.17;1.55], *p* < 0.001). Other factors associated with increased odds of LBW were substance abuse (OR = 1.60 [1.07;2.32], *p* = 0.016), if the baby was a twin (OR = 3.33 [2.39;4.54], *p* < 0.001), gestational diabetes (OR = 1.21 [1.00;1.45], *p* = 0.045), smoking at delivery (OR = 2.33, [1.93;2.82], *p* < 0.001), missing more than four antenatal appointments (OR = 1.29 [1.04;1.58], *p* = 0.019), and consanguineous union (OR = 1.27 [1.03;1.55], *p* = 0.025). Having a BMI>35 was associated with reduced odds of LBW (OR = 0.54 [0.43;0.67], *p* < 0.001).

The odds of premature birth were increased for Bangladeshi (OR = 1.25 [1.00;1.53], *p* = 0.043) and Pakistani (OR = 1.22 [1.09;1.37], *p* < 0.001) women. Women of unknown ethnicity had the greatest increase in odds of premature birth (OR = 2.90 [2.02;4.09], *p* < 0.001). Other ethnicity women had reduced odds of premature birth (OR = 0.53 [0.27;0.93], *p* < 0.042). Living in one of the 20% most deprived areas nationally was associated with increased odds of premature birth (OR = 1.12 [1.02;1.24], *p* = 0.021). Other factors associated with increased odds of premature birth were substance abuse (OR = 1.43 [1.07;1.87], *p* = 0.012), social services involvement (OR = 1.35 [1.19;1.52], *p* < 0.001), maternal age between 30 and 39 (OR = 1.12 [1.03;1.21], *p* = 0.005) or 40 and above (OR = 1.48 [1.25;1.73], *p* < 0.001), if the baby was a twin (OR = 27.2 [22.5;33.0], *p* < 0.001), mental health issues (OR = 1.15 [1.05;1.26], *p* = 0.002), sensory/physical disability (OR = 1.51 [1.29;1.74], *p* < 0.001), gestational diabetes (OR = 1.36 [1.21;1.52], *p* < 0.001), smoking at delivery (OR = 1.51 [1.33;1.71], *p* < 0.001), folic acid not being taken during pregnancy (OR = 1.15 [1.05;1.27], *p* = 0.003), and late booking of the first antenatal appointment (OR = 1.74 [1.53;1.97], *p* < 0.001).

The odds of stillbirth were greater for Black (OR = 1.75 [1.09;2.74], *p* = 0.017) or unknown ethnicity (OR = 5.28 [1.76;12.8], *p* < 0.001) women. Living in one of the 20% most deprived areas nationally was also associated with increased odds of stillbirth (OR = 1.72 [1.14;2.67], *p* = 0.013). Other factors associated with increased odds of stillbirth were smoking at delivery (OR = 1.60 [0.99;2.51], *p* = 0.046), and late booking of the first antenatal appointment (OR = 1.73 [1.07;2.67], *p* = 0.019).

The odds of neonatal death were greater for Asian (OR = 1.90 [1.38;2.63], *p* < 0.001) and unknown ethnicity (OR = 4.87 [1.96;10.7], *p* < 0.001) women. Other factors associated with increased odds of neonatal death were maternal age of 40 and above (OR = 1.82 [1.05;2.98], *p* = 0.024), if the baby was a twin (OR = 5.58 [3.05;9.41], *p* < 0.001), folic acid not being taken during pregnancy (OR = 1.98 [1.44;2.70], *p* < 0.001), and late booking of the first antenatal appointment (OR = 1.59 [1.02;2.41], *p* = 0.034).

## 4 Discussion

The present study provides a comprehensive analysis of the associations between a range of socioeconomic, clinical, and behavioural factors with key maternal outcomes among a representative cohort of BSol's maternity patients. To the author's knowledge, this is the first quantitative study to investigate the wider determinants of maternity outcomes at a local level. Ethnicity was found to be a significant risk factor for all outcomes studied. Additionally, social deprivation was associated with increased odds of LBW and stillbirth. Substance abuse, smoking at delivery, and late antenatal booking were also found to be associated with increased odds of adverse pregnancy outcomes.

A large proportion of premature births and LBWs were found to be associated with ethnic and socioeconomic inequalities. This is consistent with the findings from a UK-based study using national data which found South Asian, Black, and Mixed and other ethnicity mothers to have increased rates of preterm birth and fetal growth restriction compared to White mothers. These rates were greater still for mothers living in more deprived areas (12). The magnitudes of the attributable fractions found in this study are generally slightly lower than those found by Jardine et al. (12). This is likely due to the difference in reference groups. Due to the small number of affluent areas in BSol, this study defined the reference group as White women in the 60% least deprived areas (quintiles 3, 4 and 5) as opposed to the 20% least deprived used by Jardine et al. (12). This may also explain why the associations between maternity outcomes and IMD were found to be less pronounced in the present study. The influence of IMD was more significant for White women. Rescaling deprivation indices for local contexts would allow for greater differentiation between areas that have a similar level of deprivation on a national scale.

The association between ethnicity and each of the adverse pregnancy outcomes was further highlighted by the multiple logistic regression analyses. A meta-analysis of over 2,198,655 pregnancies in high-income and upper-middle income countries found that babies born to Black women had an increased risk of premature birth, stillbirth, neonatal death, and having a small birth weight for their gestational age (21). Babies born to South Asian women were found to have an increased risk of preterm birth and having a small birth weight for their gestational age (21). The effect of ethnicity on preterm birth and small-for-gestational-age babies was not found to vary significantly across region (21), however, Asian and Black women in the UK have also been found to have an increased risk of stillbirth (12). While the present study did not find significantly increased odds of premature birth for Black mothers or stillbirth for Asian mothers, the effect of ethnicity was largely consistent with the existing literature.

There are several potential mechanisms that explain why ethnicity is associated with increased odds of adverse pregnancy outcomes. One possible explanation is poor communication between women in maternal care and healthcare professionals and mistreatment due to discrimination. An evidence synthesis of 26 UK-based studies on the experiences of Black, Asian and minority ethnic women's experiences in maternity care found that staff, midwives and doctors communicated in a manner that was not accessible to women from minoritised ethnic groups. Women also reported differential treatment from staff including direct discrimination, stereotyping and racist comments (22). A 2010 national survey of women's experience of maternity care corroborates these findings (23). It found that Asian, Black, Mixed, and other ethnicity women were less likely to feel like staff had communicated with them well, trusting staff in providing care during labour and birth, and feeling satisfied with the care they received during labour, birth, and after birth (23). Additionally, some of the difference in fetal growth may be due to genetics. Attempts have been made to construct ethnicity-specific growth charts (24, 25). However, it is difficult to determine how much of this variation is due to confounding with other wider determinants of health. One retrospective cohort study found at least partial mediation of the difference by variation in the adequacy of prenatal care (26).

Women with an unknown ethnicity had the highest odds of premature birth, stillbirth, and neonatal death. This missing ethnicity data may be due to limited engagement of these women with the maternal healthcare system. This is supported by Figure S8 which shows that women with an unknown ethnicity made their first antenatal booking late (after 19 weeks gestation) in 42.6% of pregnancies compared to the average of 6.9%. This highlights the need for the ICS, including local authorities, to develop mechanisms that facilitate the earlier connection of these women to support that could reduce their odds of adverse birth outcomes. Such interventions will require further understanding of the underlying causes of missing demographic data and the factors contributing to its likelihood.

Results from the logistic regression reinforced the importance of IMD on pregnancy outcomes. Women in the most deprived quintile had significantly increased odds of premature birth and stillbirth. This is consistent with national studies on the association between IMD and adverse pregnancy outcomes (4, 6, 12). Financial and housing issues were associated with an increased odds of LBW and premature birth. Poverty and social deprivation are associated with a variety of negative health-related determinants and behaviours (27). A 2021 systematic review of socioeconomic inequalities and adverse pregnancy outcomes in the UK and the Republic of Ireland outlined a range of potential causal pathways for deprivation to impact pregnancy outcomes (5). These include inadequate dietary patterns (28), reduced levels of physical activity (29), poor engagement with maternal care (30), increased drug and alcohol use during pregnancy (31, 32), and a higher chance of exposure to domestic violence (33). There is also evidence that individuals experiencing economic hardship are more likely to focus on coping in the short term rather than making lifestyle changes to improve their long-term wellbeing (34). Neighbourhood socioeconomic deprivation has also been associated with increased levels of the stress hormone, cortisol (35). Deregulation of cortisol can impact metabolic regulation (36), reduce immune functions (37), and promote mental health disorders (38). Cortisol may, therefore, provide a mechanism by which deprivation can more directly impact physical health.

Supplementary Section 3.4 explores trends in risk factor prevalence, highlighting differences across ethnic and socioeconomic groups. The analysis indicates that Asian, Black, and Mixed-ethnicity women were disproportionately likely to live in the most deprived areas. The analysis also highlights how ethnicity and IMD are both associated with risk factors that significantly increase the odds of adverse pregnancy outcomes. While individual variables contribute to the odds of adverse pregnancy outcomes, their interplay may amplify or mediate these effects. Therefore, understanding these correlations provides important context for interpreting observed inequalities in adverse pregnancy outcomes.

Maternal smoking during pregnancy was associated with increased odds of LBW, premature birth, and stillbirth. The negative impact of smoking during pregnancy on fetal health has been well documented (39–41). Physical mechanisms for this include the restriction of placental blood vessels by nicotine (42) and the binding of carbon monoxide to fetal haemoglobin (43). However, studies have shown that early smoking cessation during pregnancy can significantly reduce the risk of negative outcomes (44–46). As demonstrated in Supplementary Section 3.4, the prevalence of smoking during pregnancy varies markedly across ethnicity and IMD. In agreement with previous studies, White women in the most deprived areas are found to have the highest prevalence (47, 48).

Maternal age also emerged as a significant factor, with women in the age ranges 30-39 and 40+ having an increased odds of premature birth. Additionally, women aged 40+ were found to have an increased odds of neonatal death. Advanced maternal age is a well-established risk factor for adverse birth outcomes. Part of this risk is believed to be due to a combination of the increased prevalence of comorbidities such as hypertension and diabetes, and the higher risk of medical complications (49, 50). The percentage of women in Birmingham aged 40 and over has consistently increased from 3.4% in 2014 to 4.7% in 2021 (51). The association between maternal age and adverse pregnancy outcomes is therefore increasingly important. However, the overall impact of these changing demographics is yet to be fully understood (52).

A BMI greater than 35kg/m^2^ was associated with reduced odds of LBW but no association was found for premature birth. A 2010 systematic review and meta-analysis similarly found reduced odds of LBW among overweight and obese women (53). However, the association with LBW is still contested as the protective effect disappeared after correcting for reporting bias. The study also found that, although there was no association observed for overall preterm birth, the odds of induced preterm birth was greater for overweight and obese women. Maternal obesity is also associated with increased odds of gestational diabetes (54), which the present study found to be associated with increased odds of LBW and premature birth. Another systematic review found gestational diabetes to be associated with an increased risk of macrosomia, being born large for gestational age, preeclampsia, and cesarean delivery (55). It is therefore possible that the protective effect of obesity masks comorbidities that are associated with adverse maternal outcomes.

The presence of twins was associated with significantly increased odds of LBW, premature birth, and neonatal death. Developmental growth studies have found twins to have a lower mass at the same gestation length than single-born infants (56). The association between twins and premature birth could be, in part, related to iatrogenic (induced) preterm deliveries due to the increased risk of maternal and fetal complications (57).

Consanguineous union was associated with increased odds of LBW. This is consistent with a 2017 meta-analysis which found a similar association between consanguinity and LBW (58). The analysis included many studies from South Asia and the Middle East. This is important since Asian and Middle Eastern mothers in the present study had the highest prevalence of consanguineous union (see Supplementary Figure S8). The meta-analysis did not, however, include any studies from the UK. The present study therefore provides new knowledge on consanguinity and LBW in a UK context. Consanguinity is also of particular concern due to its association with a range of negative health outcomes, particularly congenital anomalies (59).

### 4.1 Policy implications

On a global level infant mortality is identified as a priority by various organisations and supported by a range of policy and guidance documents. WHO (60) and UNICEF (61) both provide guidance on how infant mortality can be reduced. Infant mortality forms a priority for the Millennium Development Goals which include, breastfeeding, complementary feeding, routine immunisation and micronutrient supplementation. The Royal College of Paediatrics and Child Health has provided policy recommendations for England. These include: the implementation of the recommendations from the National Maternity Review (62), the implementation of the National Strategy for Child Health and Wellbeing, renewed investment and resources to support the Healthy Child Programme, and the implementation of the commitments of the NHS England long-term plan (63) as well as the Neonatal Critical Care Review (64).

The findings of this study highlight the need for targeted interventions. Resources should be focused on the most deprived and ethnically diverse areas as these women have the greatest odds of adverse pregnancy outcomes. Prenatal care plans should be sensitive to the age of the woman and the presence of sensory/physical disabilities with risk assessments conducted for those with increased risks of negative outcomes. Further study of the demographic and socioeconomic trends in risk factors and pregnancy outcomes is needed. These studies should be place-based and consider the unique populations living in different areas.

### 4.2 Strengths and limitations

This research uniquely focuses on the local context of Birmingham and Solihull, providing a nuanced understanding of the determinants directly impacting women and their babies in this location. The large size of the maternity dataset means that results are representative of the BSol maternity population attending one of the two NHS Trusts included. The high-quality maternity dataset, with 95.5% ethnicity completeness [compared to 89% for NHS Digital Maternity Services Dataset (65)] also minimises biases related to missing data and allows for a detailed analysis. The study considers detailed ethnic categories rather than broad aggregates to reflect the diverse lived experiences within these groups (66). However, broader aggregation was necessary for certain analyses due to small outcome occurrences.

This study has limitations that should be acknowledged. Firstly, the exclusion of data from City Hospital (accounting for 18% of BSol births in 2021) may limit the complete representation of BSol women in maternity care. Additionally, the IMD is an area-based aggregate measure of socioeconomic deprivation. This may lead to non-differential misclassification, diluting the true impact of socioeconomic deprivation. The sex of the babies born was also not included in the data and, therefore, the tendency of female babies to have a birth weight slightly lower than males is not considered (67). However, since the proportion of male and female babies is expected to be fairly even across the variables of interest, this is not a significant concern for this study. Only smoking status at birth was available, limiting analysis of smoking's impact during pregnancy and thus not capturing the complete impact on pregnancy outcomes. Finally, ORs have been presented for a large number of variables, thus increasing the risk of type-I error. However, since this is descriptive study, α was not reduced to avoid missing real effects.

## 5 Conclusions

This study highlights several key factors associated with adverse pregnancy outcomes in the Birmingham and Solihull area. Ethnicity emerged as a significant determinant, with various ethnic groups, including Bangladeshi, Indian, Pakistani, Asian Other, Black African, Black Caribbean, and Mixed ethnicity women, facing increased odds of negative pregnancy outcomes. The odds of premature birth, stillbirth, and neonatal death were highest among women of unknown ethnicity, emphasising the critical need for accurate data collection and reporting in healthcare systems.

The influence of financial and housing challenges on LBW emphasises the relevance of addressing socioeconomic disparities to improve pregnancy outcomes. Maternal age, the presence of twins, sensory/physical disabilities, gestational diabetes, and folic acid not being taken were also found to increase the odds of adverse pregnancy outcomes. While this study offers valuable insights into a core UK city, its findings likely reflect broader national trends, reinforcing the significance of targeted interventions and comprehensive support systems for diverse maternal populations. These findings call for further research and, more urgently, for the development of tailored public health policies and interventions to address the identified determinants of adverse pregnancy outcomes and ultimately enhance maternal and child health in Birmingham, Solihull and other areas of a similar demographic and socioeconomic population profile.

## Data Availability

The data analysed in this study is subject to the following licenses/restrictions: the raw data used in this study, although pseudonymized, is potentially identifiable. It can therefore not be made public due to confidentiality reasons. Requests to access these datasets should be directed to Birmingham Women and Children's Hospital NHS Foundation Trust, bwc.accesstohealthrecords@nhs.net.

## Footnotes

^1^England and Wales Core Cities include Birmingham, Bristol, Cardiff, Leeds, Liverpool, Manchester, Newcastle, Nottingham, and Sheffield.

^2^BSol localities: North, South, East, West, and Solihull.
