# Supplementary material for: Wider determinants of adverse birth outcomes in Birmingham and Solihull
Source: Front Public Health. 2025 Apr 15;13:1544903. doi: 10.3389/fpubh.2025.1544903 (PMC12037573; doi:10.3389/fpubh.2025.1544903)
Supplement: Supplementary file 1 [file Presentation_1.pdf]

Supplementary Material

1 TRENDS IN PREGNANCY OUTCOMES

As shown in Figure. S1, the crude rates of low birth weight (LBW), premature birth, stillbirth, neonatal mortality, and infant mortality are all significantly higher in Birmingham and Solihull (BSol) than the national average and have been for at least 10 years. However, it can be seen that the rates for BSol are dominated by the Birmingham contribution and that Solihull is often much closer to the national average and, for stillbirth, significantly below it OHID (2024).

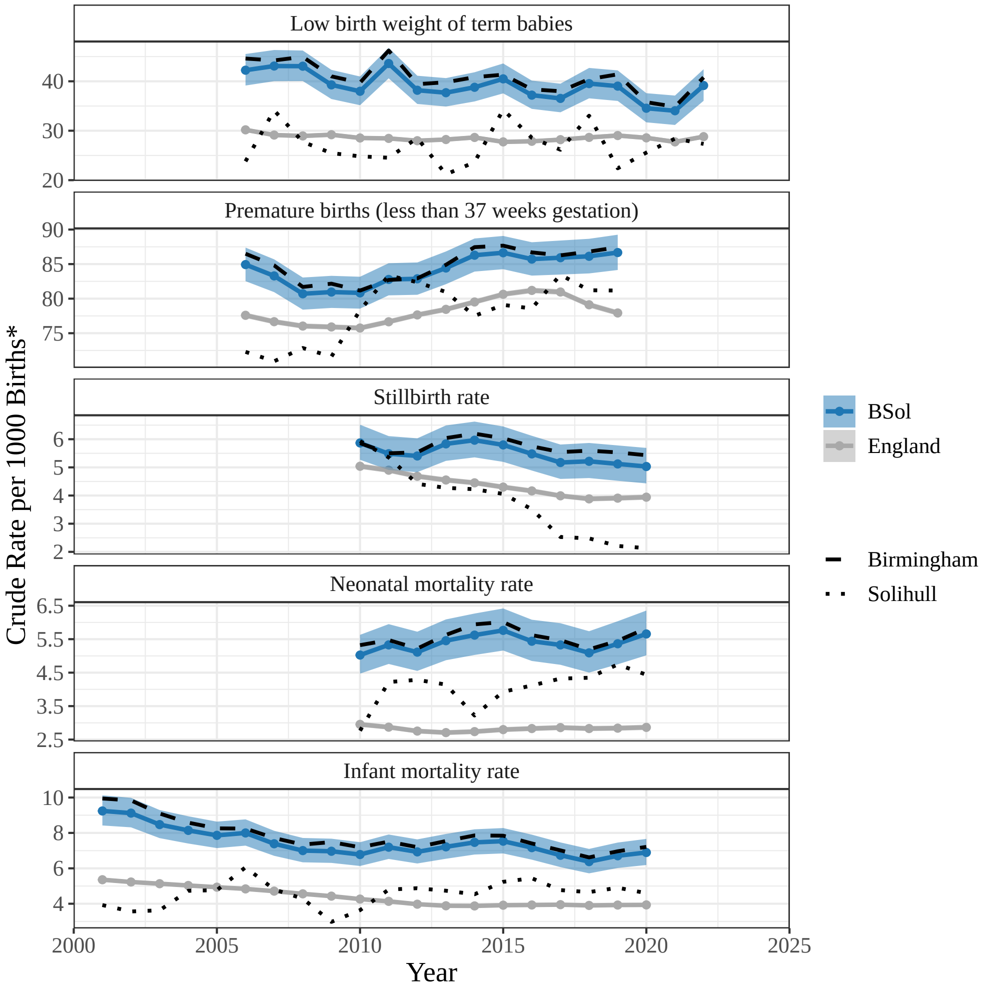

Figure S1. Crude rates of LBW of term babies, premature birth, stillbirth, neonatal mortality, and infant mortality per 1000 births. *The denominators are the number of live births at term for LBW, the number of live births and stillbirths for premature birth and stillbirth, and the number of live births for neonatal and infant mortality. The blue and grey shaded regions show the 95% CI calculated using the Byar method for Bsol and England respectively OHID (2024).

In 2022, the rate of low birth weight term babies per 1000 live births at term was 39.1 (95%CI: 36.1, 42.4) in BSol (Birmingham: 40.8, Solihull: 27.3). This is 39% higher than the England average of 28.8 (28.3, 29.3). Overall, the LBW rate in BSol has fallen slightly quicker than the national average from 42.2 (39.1, 45.5) in 2006. The Solihull rate, however, has remained consistently much closer to the national rate. Combined, the rate in BSol has been consistently significantly higher than in England.

In 2019-2021, the rate of premature births per 1000 live births and stillbirths was 86.7 (84.1, 89.3) in BSol (Birmingham: 87.4, Solihull: 81.2). This is 11% higher than the England average of 77.9 (77.5, 78.3). The premature birth rate fell in BSol and England from 2006-08 until 2008-2010 for BSol and 2010-2012 for England. The rate then increased in England until around 2016-2018 but has fallen every year since. The rate in BSol also increased until around 2015-2017 when it plateaued. Solihull has not followed this general trend but has mostly had a premature birth rate much lower than Birmingham. Combined, the rate in BSol has been consistently significantly higher than in England.

In 2020-2022, the stillbirth rate per 1000 live and stillbirths was 5.03 (4.43, 5.69) in BSol (Birmingham: 5.42, Solihull: 2.13). This is 28% higher than the England average of 3.94 (3.85, 4.04). The stillbirth rate in England has fallen every year from 5.04 (4.94, 5.14) in 2010-2012. In BSol, the stillbirth rate has also been generally decreasing, however, there was a two-year rise from 2012-14 to a peak rate of 5.97 (5.36, 6.63) 2014-16. The stillbirth rate in Solihull has also fallen every year but much more quickly than in England or Birmingham. The rate in BSol has been significantly higher than in England almost every year.

In the same period, the neonatal mortality rate per 1000 live births was 5.66 (5.02, 6.36) in BSol (Birmingham: 5.82, Solihull: 4.44). This is 98% higher than the England average of 2.86 (2.79, 2.95). The neonatal mortality rate in England has changed very little from 2010-2012 when it was at 2.95 (2.88, 3.03). In BSol, the rate has increased from 5.03 (4.47, 5.63) in 2010. However, Solihull has experienced the the largest increase in neonatal mortality rate from 2.77 (1.66, 4,32) in 2010-2012. The rate in BSol has been consistently significantly higher than in England.

Also in 2020-2022, the infant mortality rate per 1000 live births was 6.90 (6.19, 7.66) in BSol (Birmingham: 7.21, Solihull: 4.60). This is 76% higher than the England average of 3.93 (3.84, 4.03). Overall, the infant mortality rates in both England and BSol have fallen since 2001-2003 from 5.36, (5.25, 5.47), and 9.24 (8.42, 10.12) respectively. However, the rate in BSol has been consistently significantly higher than in England.

2 VARIANCE INFLATION FACTOR

The variance inflation factor (VIF) serves as a critical diagnostic tool to assess multicollinearity among the independent variables in the regression models. Multicollinearity, the high correlation between two or more predictor variables, can undermine the reliability and interpretability of regression results by inflating standard errors and making it challenging to discern the individual contributions of each variable. By calculating and examining the VIF for each independent variable, multicollinearity issues can be identified and addressed, thus ensuring the robustness and accuracy of the regression analyses. This is particularly important in this study, due to the complex interplay of various risk factors contributing to adverse birth outcomes, and precise estimation of their effects is paramount for informed public health interventions and policy recommendations.

It can be seen from Figure. S2 that all of the predictor variables have VIF values well below the typical threshold value of 5.

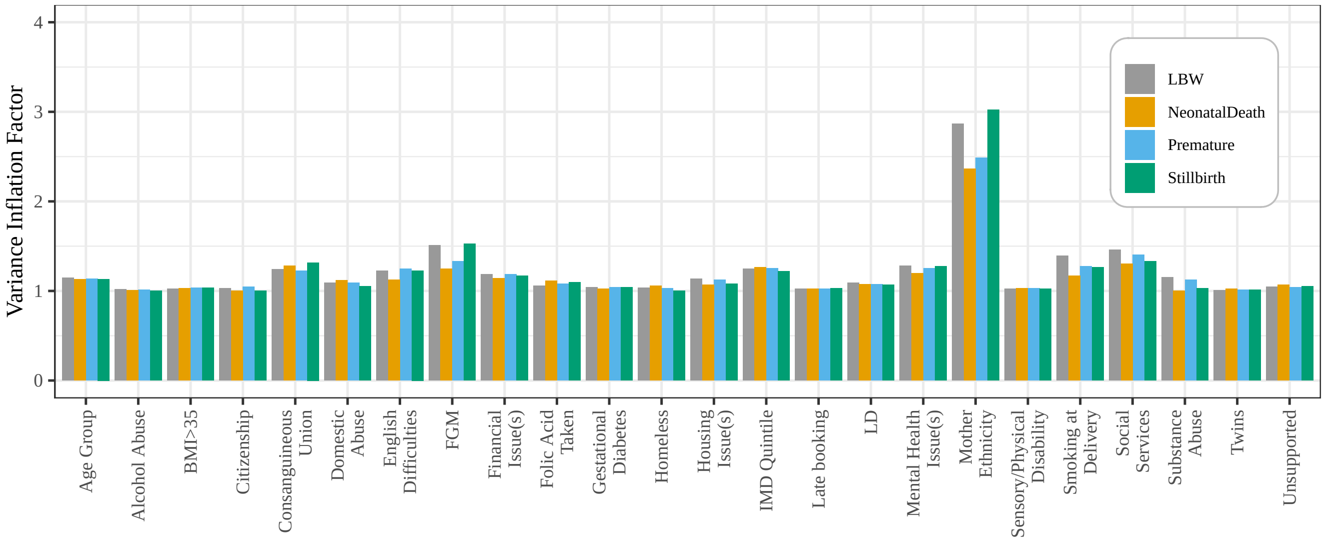

Figure S2. Variance inflation factor for each of the outcomes studied. Calculated using the `carat` package in R Kuhn (2008).

3 POPULATION ANALYSIS

3.1 Geography

After data cleaning and exclusion, the Birmingham Central locality had the highest number of births (N=7654 or 19.1%), followed by Birmingham West (N=6577 or 16.5%), Birmingham East (N=5735 or 14.3%), Birmingham South (N=4967 or 12.4%), Birmingham North (N=4818 or 12.1%) and Solihull (N=4277 or 10.7%). The distribution of births across these localities is shown in Figure. S3. There were an additional 5943 births recorded as ”Non-geographic” (14.9%) and one recorded as ”Unknown”. The locations of each maternity ward included in the data are also shown.

3.2 Outcome Distribution

Table. S1 gives the combinations of intermediate outcomes (Full term normal birth weight (NBW), LBW, and premature birth) and final outcomes (Postnatal, stillbirth, and neonatal death) for births in the data after exclusion. We see that most (82%) of the babies who died during the neonatal period were premature. None of the stillborn babies were recorded as LBW or premature.

Table S1. Intersection of intermediate outcomes (Full-term normal birth weight (NBW), LBW, and premature birth) and final outcomes (Postnatal, stillbirth, and neonatal death).

	Full Term NBW	LBW	Premature
**Postnatal**	35257	1188	3141
**Neonatal Death**	28	9	171
**Stillbirth**	178	0	0

Tables. S2 and S3 give a breakdown of each explanatory variable across intermediate and final variables respectively.

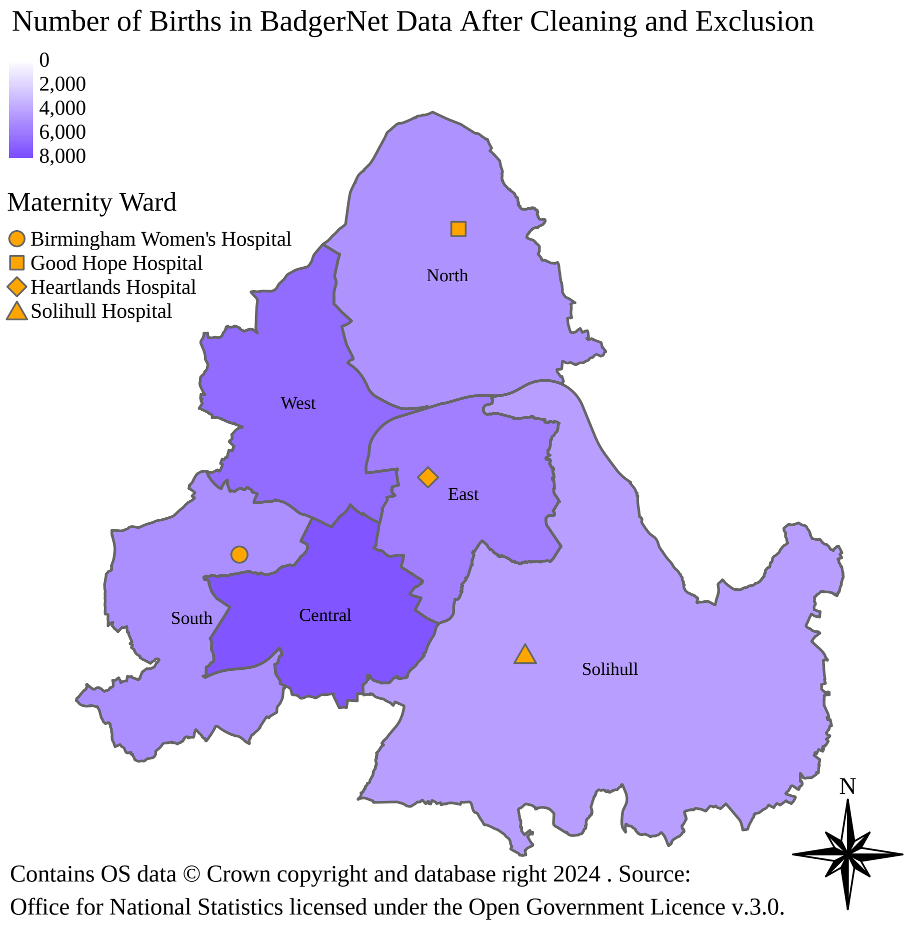

Figure S3. Distribution of births in the final BadgerNet data set where the address of the woman was known. The R code used for map plotting is available on GitHub: BSol.mapR.

Table S2. Breakdown of intermediate birth outcomes (normal, LBW, and premature birth) across different demographic and medical factors. Counts less than or equal to five have been suppressed. Table produced using R package `gtsummary` Sjoberg et al. (2021).

**Variable**	**Normal**, N = 35,463	**LBW**, N = 1,197	**Premature**, N = 3,312
**Mother Ethnicity**			
Asian-Other	1,106 (3.1%)	46 (3.8%)	93 (2.8%)
Bangladeshi	1,044 (2.9%)	62 (5.2%)	114 (3.4%)
Black-Other	333 (0.9%)	11 (0.9%)	23 (0.7%)
Black African	2,319 (6.5%)	70 (5.8%)	191 (5.8%)
Black Caribbean	808 (2.3%)	43 (3.6%)	81 (2.4%)
Chinese	252 (0.7%)	10 (0.8%)	17 (0.5%)
Eastern European	700 (2.0%)	17 (1.4%)	62 (1.9%)
Indian	1,783 (5.0%)	83 (6.9%)	148 (4.5%)
Irish	167 (0.5%)	$\leq$5	9 (0.3%)
Middle Eastern	510 (1.4%)	21 (1.8%)	38 (1.1%)
Mixed-Other	305 (0.9%)	16 (1.3%)	24 (0.7%)
Other	242 (0.7%)	5 (0.4%)	12 (0.4%)
Pakistani	7,179 (20%)	331 (28%)	737 (22%)
Unknown	153 (0.4%)	4 (0.3%)	59 (1.8%)
White and Asian	204 (0.6%)	13 (1.1%)	16 (0.5%)
White and Black African	97 (0.3%)	$\leq$5	11 (0.3%)
White and Black Caribbean	594 (1.7%)	27 (2.3%)	72 (2.2%)
White British	15,411 (43%)	391 (33%)	1,435 (43%)
White-Other	2,256 (6.4%)	42 (3.5%)	170 (5.1%)
**IMD Quintile**			
1	19,089 (54%)	747 (62%)	1,960 (59%)
2	6,551 (18%)	202 (17%)	540 (16%)
3+	9,823 (28%)	248 (21%)	812 (25%)
**Age Group**			
Less than 20	888 (2.5%)	39 (3.3%)	96 (2.9%)
20-29	14,588 (41%)	506 (42%)	1,284 (39%)
30-39	18,350 (52%)	592 (49%)	1,714 (52%)
40+	1,637 (4.6%)	60 (5.0%)	218 (6.6%)
**Twins**	121 (0.3%)	44 (3.7%)	347 (10%)
**Financial/housing issues**	6,952 (20%)	348 (29%)	807 (24%)
**Substance Abuse**	318 (0.9%)	34 (2.8%)	72 (2.2%)
**Social Services**	3,211 (9.1%)	174 (15%)	489 (15%)
**Mental health issue(s)**	9,357 (26%)	354 (30%)	1,032 (31%)
**Sensory/physical disability**	1,622 (4.6%)	58 (4.8%)	230 (6.9%)
**BMI>35**	4,569 (13%)	90 (7.5%)	438 (13%)
**Gestational Diabetes**	3,425 (9.7%)	140 (12%)	422 (13%)
**Smoking at delivery**	2,623 (7.4%)	190 (16%)	419 (13%)
**Folic Acid Taken**	29,417 (83%)	967 (81%)	2,611 (79%)
**Late antenatal booking**	2,298 (6.5%)	101 (8.4%)	376 (11%)
**>4 missed appointments**	1,892 (5.3%)	108 (9.0%)	211 (6.4%)
**Consanguineous Union**	2,532 (7.1%)	132 (11%)	250 (7.5%)

Table S3. Breakdown of final birth outcomes (normal, stillbirth, and neonatal death) across different demographic and medical factors. Counts less than or equal to five have been suppressed. Table produced using R package `gtsummary` Sjoberg et al. (2021).

**Variable**	**Normal**, N = 39,586	**Stillbirth**, N = 178	**Neonatal Death**, N = 208
**Ethnicity Group**			
Asian	12,857 (32%)	53 (30%)	95 (46%)
Black	3,835 (9.7%)	29 (16%)	15 (7.2%)
Middle Eastern	566 (1.4%)	≤5	≤5
Mixed	1,369 (3.5%)	7 (3.9%)	≤5
Other	257 (0.6%)	0 (0%)	≤5
Unknown	203 (0.5%)	≤5 (2.8%)	8 (3.8%)
White	20,499 (52%)	82 (46%)	83 (40%)
**IMD Quintile**			
1	21,555 (54%)	118 (66%)	123 (59%)
2	7,227 (18%)	30 (17%)	36 (17%)
3+	10,804 (27%)	30 (17%)	49 (24%)
**Age Group**			
Less than 20	1,012 (2.6%)	5 (2.8%)	6 (2.9%)
20-29	16,220 (41%)	75 (42%)	83 (40%)
30-39	20,471 (52%)	84 (47%)	101 (49%)
40+	1,883 (4.8%)	14 (7.9%)	18 (8.7%)
**Twins**	493 (1.2%)	5 (2.8%)	14 (6.7%)
**Folic acid taken**	32,719 (83%)	134 (75%)	142 (68%)
**Financial/housing issue(s)**	8,012 (20%)	47 (26%)	48 (23%)
**Smoking at delivery**	3,190 (8.1%)	25 (14%)	17 (8.2%)
**Mental health issue(s)**	10,633 (27%)	57 (32%)	53 (25%)
**Late antenatal booking**	2,722 (6.9%)	25 (14%)	28 (13%)

3.3 Demographics

As shown in Figure. S4, 52% of women identified as White, 75% of which were ”White British”. The second largest group were Asian women who made up 33% of all births in the city. 64% of these Asian women identified as Pakistani.

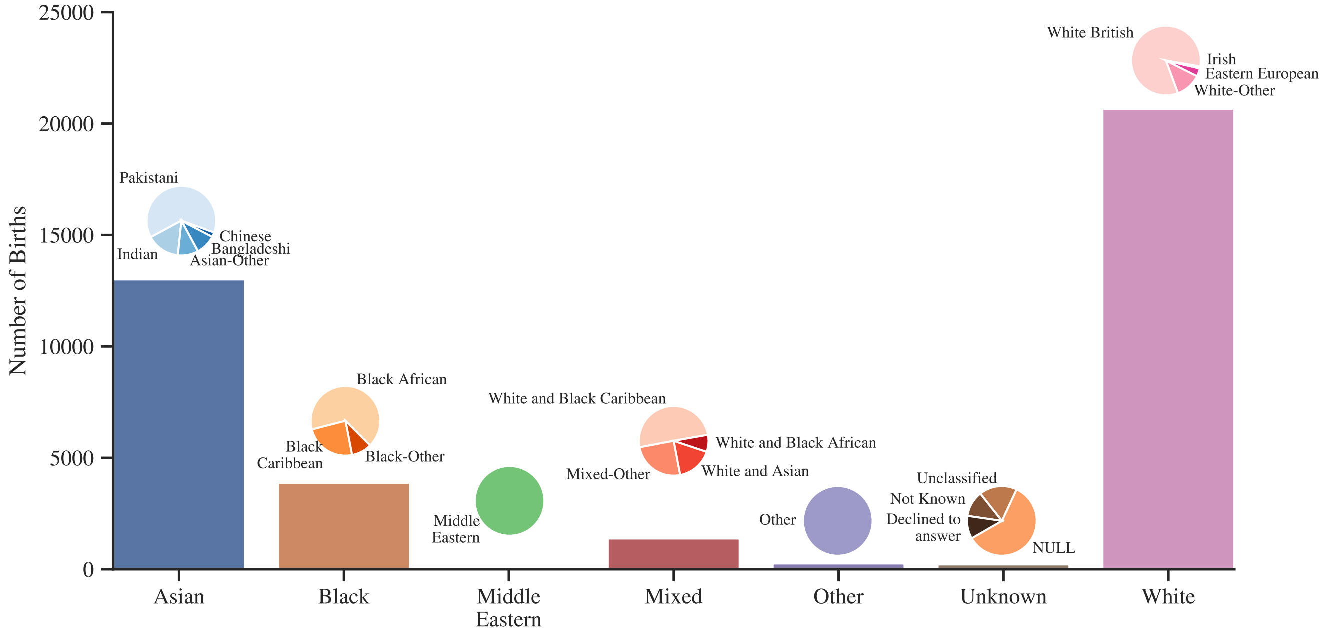

Figure S4. Ethnicity distribution of women who gave birth in Birmingham and Solihull between October 2020 and April 2023.

More than half (54.4%) of the women live in one of the 20% most deprived areas of England. However, as shown in Figure. S5, this deprivation is not experienced equally across different ethnic groups. T White women were less likely to live in the most deprived areas, with 42.7% of White women living in the most deprived areas. Meanwhile, Black women were the most likely with 73.0% living in the most deprived areas. Asian and Middle Eastern women had similar rates with 67.1% and 68.8% respectively living in the most deprived areas. Women of Mixed or Other ethnic origin lived in the most deprived areas 58.9% and 59.1% of the time respectively.

3.4 Risk Factors

The prevalence of the different risk factors studied here are given in Figures. S6, S7, and S8. All risk factor prevalences for each ethnicity-IMD combination are compared to the prevalence for White women in the least deprived areas using a two-sample t-test. The statistical significance of the absolute differences is indicated using standard asterisk notation [1].

Financial/housing issues (Figure. S6A) were recorded in $20.3 \pm 0.9\%$ of the birth episodes in the data. Those living in the most deprived quintile were predictably much more likely to have financial/housing issues ($27.1 \pm 0.6\%$) than those in the least deprived quintile ($4.8 \pm 0.8\%$). The prevalence of financial/housing issues was highest amongst Middle Eastern ($31.6 \pm 3.8\%$), Black ($31.0 \pm 1.5\%$), and Mixed ethnicity ($30.6 \pm 2.4\%$) women.

[1] 1, 2 and 3 *’s refer to $p < 0.1$, $p < 0.05$, and $p < 0.01$ respectively.

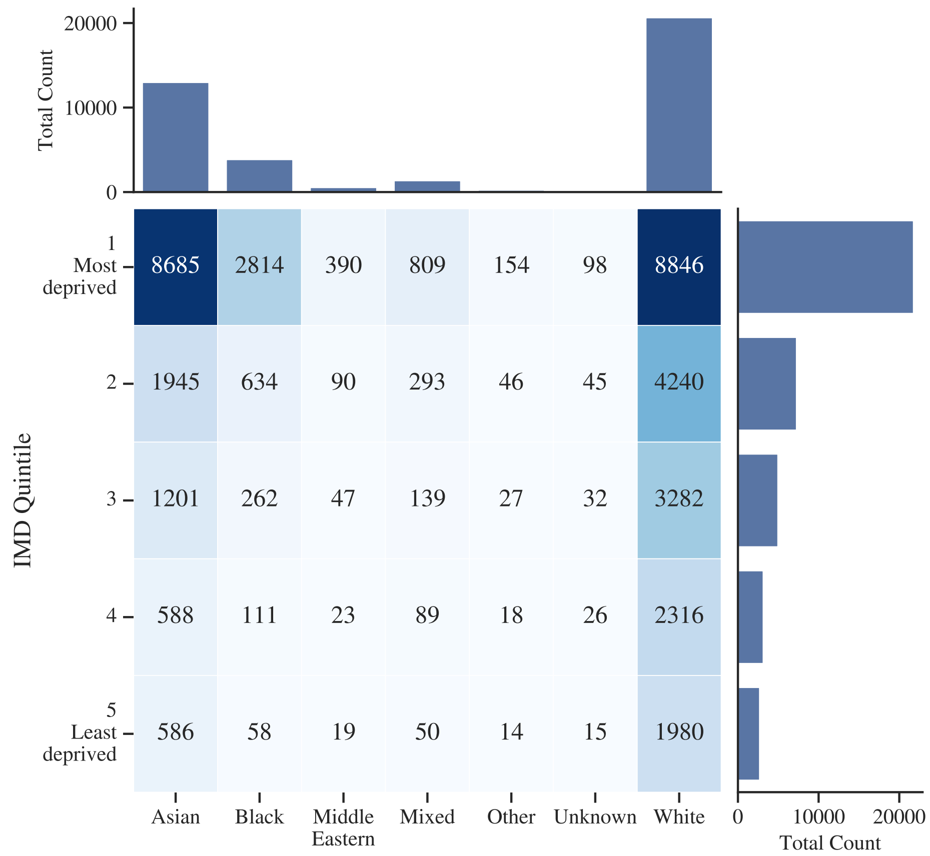

Figure S5. Distribution of women who gave birth in Birmingham between October 2020 and April 2023 across ethnic category and Index of Multiple Deprivation. Code for calculating and inequality matrices available on GitHub: EquiPy.

Social services (Figure. S6B) were involved in $9.7 \pm 0.9\%$ of birth episodes in the data. Births to women living in the most deprived quintile were much more likely to have social services involvement ($12.5 \pm 0.4\%$) than those in the least deprived quintile ($3.2 \pm 0.7\%$). The prevalence of social services involvement was highest amongst Mixed ethnicity ($18.5 \pm 2.0\%$) and White ($12.8\% \pm 0.5$) women.

Women were recorded as smoking at birth (Figure. S6C) for $8.1 \pm 0.9\%$ of birth episodes in the data. This is lower than the national value for smoking during early pregnancy ($12.7 \pm 0.1\%$ in 2018/19), however, while presumably correlated, these values do not measure the same thing OHID (2024). Women living in the most deprived quintile were much more likely to be smoking at birth ($10.3 \pm 0.4\%$) than those in the least deprived quintile ($2.1 \pm 0.5\%$). The prevalence of smoking at birth was highest amongst White ($13.1 \pm 0.5\%$) and Mixed ethnicity ($12.1 \pm 1.7\%$) women.

Substance abuse (Figure. S6D) was reported for $1.1 \pm 1.0\%$ of birth episodes in the data. This is consistent with the 2018/19 national average for drug misuse in early pregnancy at $1.4 \pm 0.1\%$ OHID (2024). Substance abuse was much more prevalent for women living in the most deprived quintile ($1.4 \pm 0.2\%$) than those in the least deprived quintile ($0.2 \pm 0.2\%$). The prevalence of substance abuse was highest amongst those with a Mixed ($2.7 \pm 0.9\%$), unknown ($1.9 \pm 1.8\%$), or White ($1.6 \pm 0.2\%$) ethnicity.

The average age of all women was 30 years. Teenage pregnancies (women aged between 12 and 17 years) (Figure. S6E) accounted for $0.7 \pm 1.0\%$ of the birth episodes in the data. This is consistent with the 2022/23

national average of 0.6% OHID (2024). Teenage pregnancies were more prevalent in the least deprived quintile ($0.9 \pm 0.1\%$) than the most deprived quintile ($0.1 \pm 0.1\%$). Women with a Mixed ($1.9 \pm 0.7\%$) or White ($1.0 \pm 0.1\%$) ethnicity had the highest percentage of teenage births.

Women aged 40+ (Figure. S6F) accounted for $4.8 \pm 1.0\%$ of birth episodes in the data. This is also consistent with the 2017 national average of 4.4% OHID (2024). Giving birth after the age of 40 or above was slightly more common in the least deprived quintile ($6.1 \pm 0.9\%$) than the most deprived quintile ($4.6 \pm 0.6\%$). Women with an Other ($9.7 \pm 3.6\%$), Black ($7.7 \pm 0.8\%$) or Middle Eastern ($7.2 \pm 2.1\%$) ethnicity had the highest percentage of births to an age of 40 or above.

Breastfeeding at initiation (Figure. S7A) was recorded for $78.8 \pm 0.5\%$ of birth episodes in the data. This is higher than the 2020/21 national average of $71.7 \pm 0.1\%$ OHID (2024). Women living in the most deprived quintile were slightly less likely to breastfeed at initiation ($76.5 \pm 0.6\%$) than women living in the least deprived quintile ($83.9 \pm 1.4\%$). Rates of breastfeeding at initiation were lowest amongst women with an unknown ($62.5 \pm 6.5\%$) or White ($72.6 \pm 0.6\%$) ethnicity.

Women were recorded as having sensory/physical disabilities (Figure. S7B) for $4.8 \pm 1.0\%$ of birth episodes in the data. Women living in the most deprived quintile had a higher prevalence of sensory/physical disabilities ($5.2 \pm 0.3\%$) than women living in the least deprived quintile ($3.3 \pm 0.7\%$). Recorded rates of sensory/physical disabilities were highest amongst women with a Mixed ($5.4 \pm 1.2\%$) or White ($5.2.6 \pm 0.4\%$) ethnicity.

Twins (Figure. S7C) were born in $1.3 \pm 1.0\%$ of birth episodes in the data. Twins were slightly more common for women living in the least deprived quintile ($5.2 \pm 0.3\%$) than the most deprived quintile ($3.3 \pm 0.7\%$). The rate of twin births was much higher for women with an unknown ethnicity ($5.1 \pm 2.9\%$). It should be noted that only the first birth of each birth episode is considered, and twins are therefore not double-counted.

One or more mental health issues (Figure. S7D) were recorded for $26.9 \pm 0.8\%$ of birth episodes in the data. There was a limited trend in recorded mental health issue prevalence across deprivation. However, the rate of mental health issues was much higher for women with a Mixed ($37.5 \pm 2.6\%$) or White (37.0 ± 0.7) ethnicity.

Women were recorded as having a BMI in the obese range (Figure. S7E) for $12.8 \pm 0.9\%$ of birth episodes in the data. This is much lower than the 2018/19 national average for obesity in early pregnancy at $22.1 \pm 0.2\%$ OHID (2024). The prevalence of obesity was highest in the most deprived quintile (14.2 ± 0.5) and lowest in the least deprived quintile (8.1 ± 1.0). Recorded obesity prevalence was highest amongst women with a Mixed (14.9 ± 1.9), White (14.6 ± 0.5), or Black (13.1 ± 1.1) ethnicity.

Women were recorded as having gestational diabetes (Figure. S7F) for $10.0 \pm 0.9\%$ of birth episodes in the data. The prevalence of gestational diabetes was highest in the most deprived quintile (11.1 ± 0.4) and lowest in the least deprived quintile (8.1 ± 1.0). Gestational diabetes prevalence was highest amongst women with an Other (14.7 ± 4.3), Asian (13.5 ± 0.6), Middle Eastern (12.5 ± 2.7) or Black (11.1 ± 1.0) ethnicity.

Folic acid was recorded as having been taken (Figure. S8A) for $82.5 \pm 0.4\%$ of birth episodes in the data. Folic acid consumption was lowest in the most deprived quintile (79.1 ± 0.5) and highest in the least deprived quintile (89.7 ± 1.1). Recorded consumption of folic acid consumption has by far the lowest for women with an unknown ethnicity ($26.9 \pm 5.9\%$). The prevalence of folic acid consumption was also lower for women with an Other (73.4 ± 5.4) or Black ($77.3 \pm 1.3\%$) ethnicity.

The first antenatal booking was made late (after 19 weeks gestation)(Figure. S8B) for $6.9 \pm 0.9\%$ of birth episodes in the data. The prevalence of late antenatal booking was slightly higher in the most deprived quintile ($7.9 \pm 0.4\%$) than in the least deprived quintile ($5.7 \pm 0.9\%$). Consistent with the early booking rate, late antenatal booking was most prevalent amongst women with an unknown ethnicity ($42.6 \pm 6.6\%$). Women with an Other ($13.5 \pm 4.2\%$), Black ($12.5 \pm 1.0\%$), or Middle Eastern ($10.7 \pm 2.5\%$) ethnicity also had comparatively high late booking prevalence.

More than four antenatal appointments (Figure. S8C) were recorded as missed for $5.5 \pm 1.0\%$ of birth episodes in the data. The prevalence of missing more than four appointments was highest in the most deprived quintile ($7.6 \pm 0.4\%$) and lowest in the least deprived quintile ($1.0 \pm 0.4\%$). The prevalence of missing more than four appointments was highest amongst women with a Black ($8.7 \pm 0.9\%$) or Mixed ($8.4 \pm 1.5\%$) ethnicity.

Consanguineous union (Figure. S8D) was recorded for $7.3 \pm 0.9\%$ of birth episodes in the data. The prevalence of consanguinity was much higher in the most deprived quintile ($11.2 \pm 0.4\%$) than in the least deprived quintile ($1.1 \pm 0.4\%$). The prevalence of consanguinity was highest amongst women with a Middle Eastern ($20.6 \pm 3.3\%$), Asian ($19.3 \pm 0.7\%$), or Other ($10.4 \pm 3.7\%$) ethnicity.

Domestic abuse (Figure. S8E) was recorded for $1.8 \pm 0.8\%$ of birth episodes in the data. The prevalence of domestic abuse was highest in the most deprived quintile ($2.2 \pm 0.2\%$) and lowest in the least deprived quintile ($0.7 \pm 0.3\%$). The prevalence of domestic abuse was highest amongst women with a Mixed ($3.0 \pm 0.8\%$) ethnicity.

Female genital mutilation (FGM) (Figure. S8F) was recorded for $3.2 \pm 0.8\%$ of birth episodes in the data. The prevalence of FGM was highest in the most deprived quintile ($5.1 \pm 0.2\%$) and lowest in the least deprived quintile ($0.1 \pm 0.1\%$). The prevalence of FGM was highest amongst women with a Black ($26.3 \pm 1.2\%$) or Middle Eastern ($14.1 \pm 0.8\%$) ethnicity.

REFERENCES

[Dataset] OHID. Office for health improvement and disparities. public health profiles 2024. https://fingertips.phe.org.uk (2024). © Crown copyright 2024. Accessed: 2024-03-22.

Kuhn M. Building predictive models in r using the caret package. *Journal of Statistical Software, Articles* **28** (2008) 1–26. doi:10.18637/jss.v028.i05.

Sjoberg DD, Whiting K, Curry M, Lavery JA, Larmarange J. Reproducible summary tables with the gtsummary package. *The R Journal* **13** (2021) 570–580. doi:10.32614/RJ-2021-053.

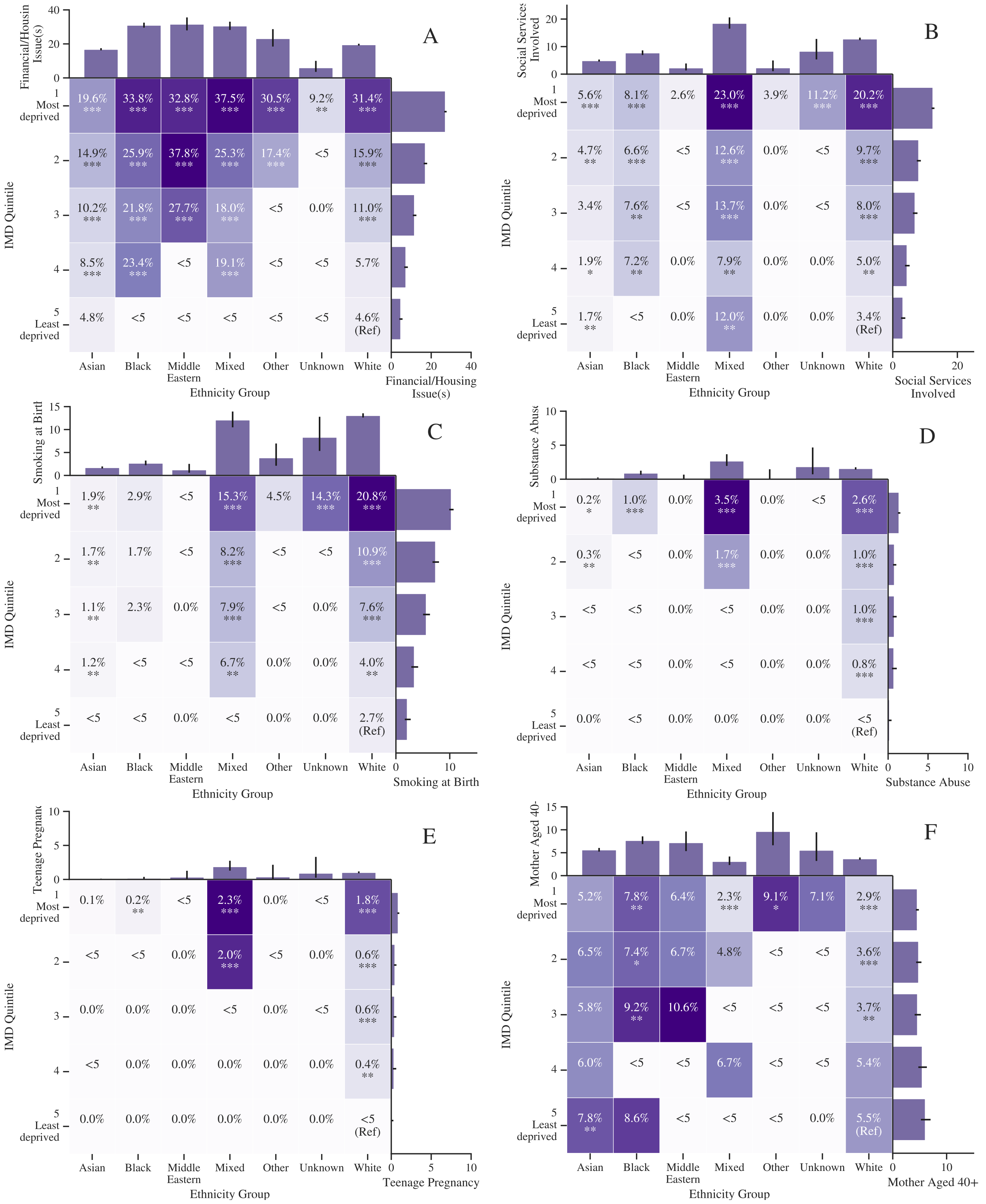

Figure S6. Risk factor prevalence for each IMD and broad ethnicity combination. A: Financial/housing issue(s), B: social services involvement, C: smoking at birth, D: substance abuse, E: teenage pregnancy, F: woman aged 40+. The error bars indicate the 95% CI calculated using the Wilson method. Numbers relating to fewer than five women have been suppressed. Code for calculating and plotting inequality matrices available on GitHub: EquiPy.

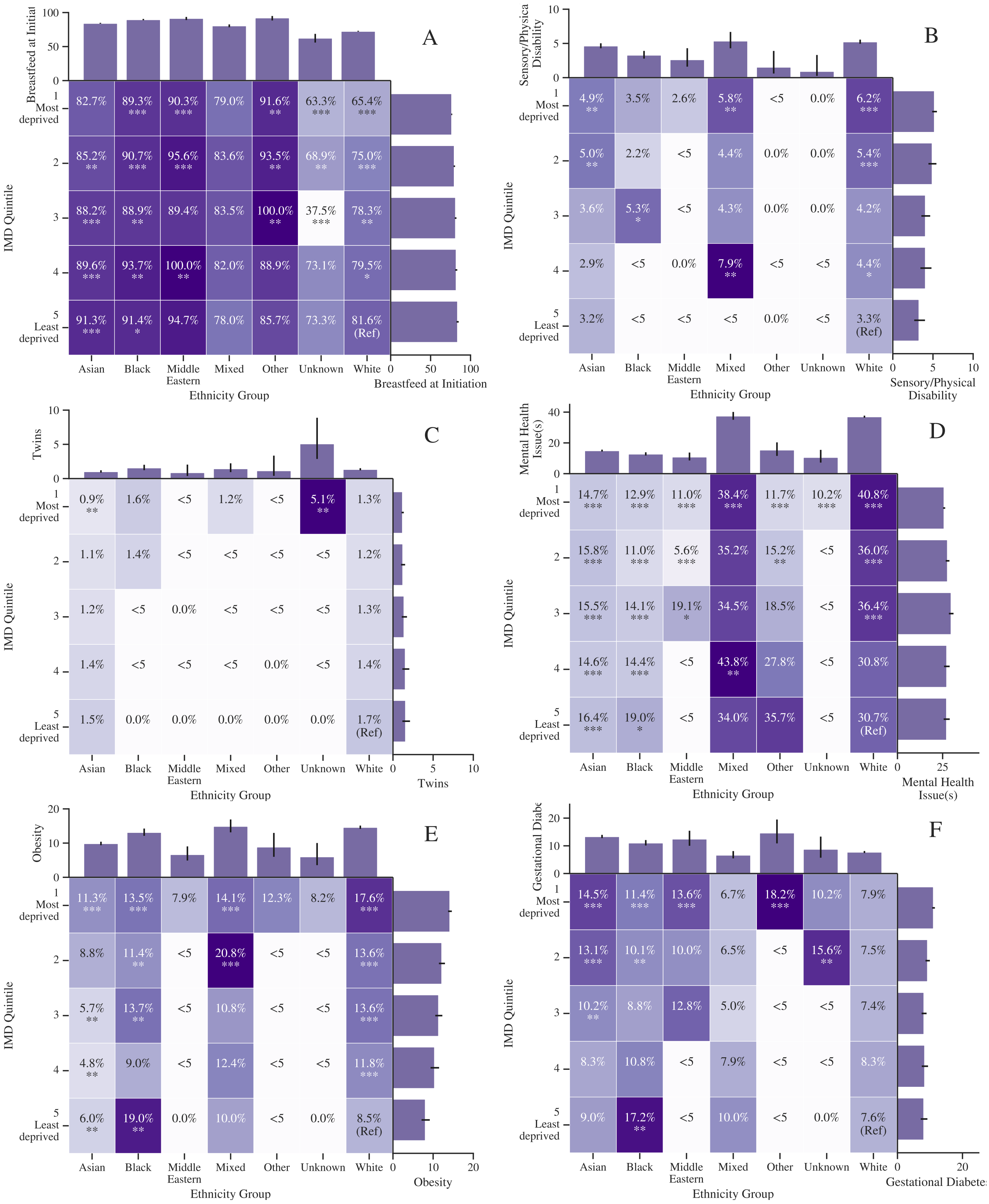

Figure S7. Risk factor prevalence for each IMD and broad ethnicity combination. A: Baby breastfed at initiation, B: Sensory/physical disability, C: twins, D: mental health issue(s), E: obesity, F: gestational diabetes. The error bars indicate the 95% CI calculated using the Wilson method. Numbers relating to fewer than five women have been suppressed. Code for calculating and plotting inequality matrices available on GitHub: EquiPy.

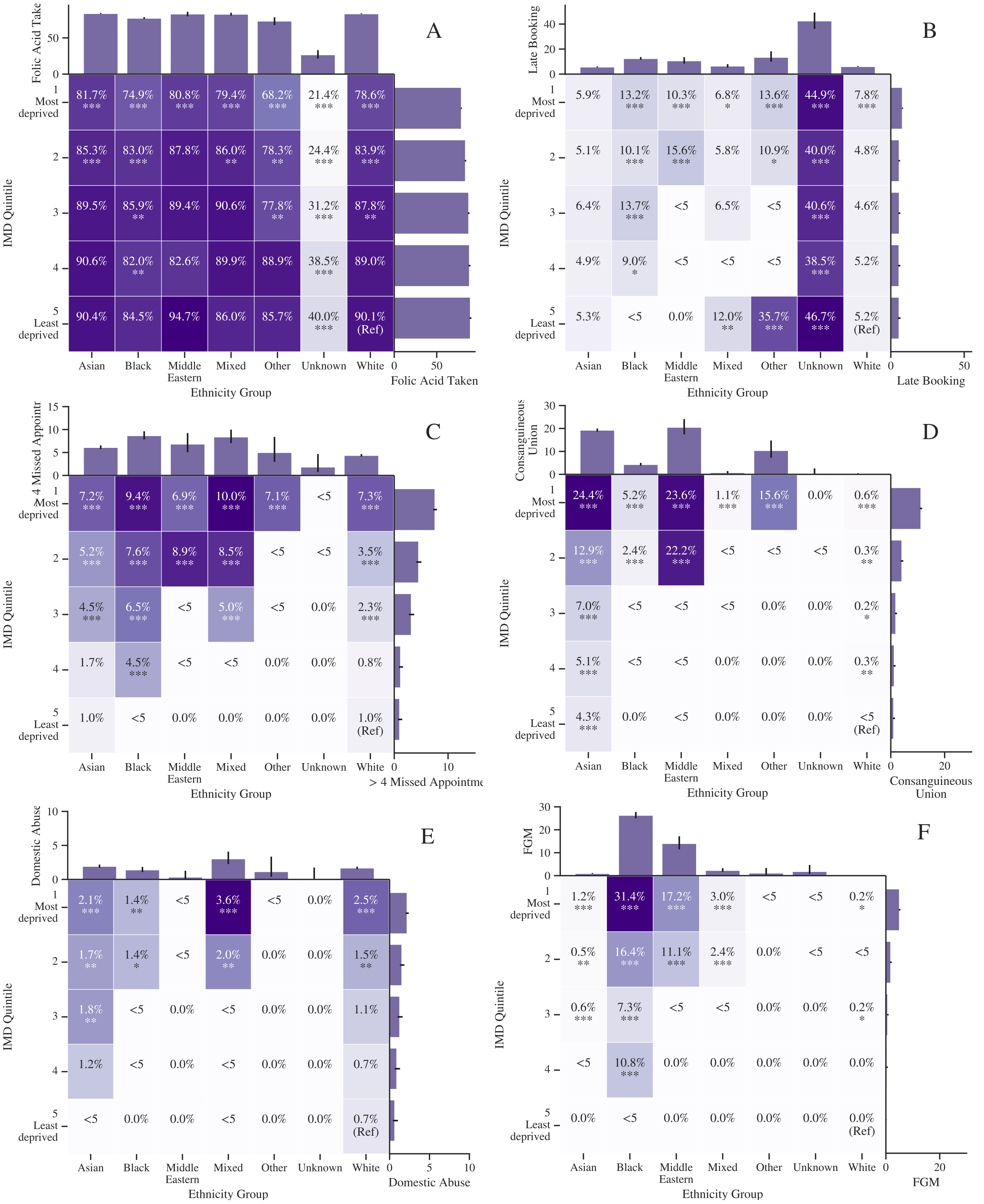

Figure S8. Risk factor prevalence for each IMD and broad ethnicity combination. A: Folic acid taken B: late antenatal booking, C: more than four antenatal appointments missed, D: consanguineous union, E: domestic abuse, F: FGM. The error bars indicate the 95% CI calculated using the Wilson method. Numbers relating to fewer than five women have been suppressed. Code for calculating and plotting inequality matrices available on GitHub: EquiPy.
